# The Protective Effect of Minocycline in a Paraquat-Induced Parkinson's Disease Model in *Drosophila* is Modified in Altered Genetic Backgrounds

**DOI:** 10.1155/2012/938528

**Published:** 2012-07-30

**Authors:** Arati A. Inamdar, Anathbandhu Chaudhuri, Janis O'Donnell

**Affiliations:** ^1^Department of Biological Sciences, University of Alabama, Tuscaloosa, AL 35487-0344, USA; ^2^Department of Plant Biology and Pathology, Rutgers University-The State University of New Jersey, Room 291D, Foran Hall, 59 Dudley Road, New Brunswick, NJ 08901, USA; ^3^Department of Pharmacology and Experimental Neuroscience, University of Nebraska Medical Center, Omaha, NE 68198-5800, USA

## Abstract

Epidemiological studies link the herbicide paraquat to increased incidence of Parkinson's disease (PD). We previously reported that *Drosophila* exposed to paraquat recapitulate PD symptoms, including region-specific degeneration of dopaminergic neurons. Minocycline, a tetracycline derivative, exerts ameliorative effects in neurodegenerative disease models, including *Drosophila*. We investigated whether our environmental toxin-based PD model could contribute to an understanding of cellular and genetic mechanisms of minocycline action and whether we could assess potential interference with these drug effects in altered genetic backgrounds. Cofeeding of minocycline with paraquat prolonged survival, rescued mobility defects, blocked generation of reactive oxygen species, and extended dopaminergic neuron survival, as has been reported previously for a genetic model of PD in *Drosophila*. We then extended this study to identify potential interactions of minocycline with genes regulating dopamine homeostasis that might modify protection against paraquat and found that deficits in GTP cyclohydrolase adversely affect minocycline rescue. We further performed genetic studies to identify signaling pathways that are necessary for minocycline protection against paraquat toxicity and found that mutations in the *Drosophila* genes that encode c-Jun N-terminal kinase (JNK) and Akt/Protein kinase B block minocycline rescue.

## 1. Introduction

The pathogenic mechanisms of PD, whether triggered by mutations or aberrant copy number of a PD-associated gene or by environmental sources, are associated with increased oxidative stress within dopaminergic neurons, exacerbated by the highly reactive nature of dopamine itself [1–3]. Chronic neuronal dysfunction overstimulates the normally protective neuroinflammatory response further amplifies oxidative conditions, accelerating disease progression [4, 5]. Therefore, compounds with antioxidant and anti-inflammatory capabilities are of great interest for their potential therapeutic benefits in chronic neurodegenerative diseases including PD. Minocycline (MC), a second generation tetracycline drug known to be clinically safe [6], has shown promising ameliorative effects in animal models for chronic neurodegenerative diseases, including neurotoxin-induced PD models [7–9]. MC appears to have anti-inflammatory properties that mediate neuroprotection in PD animal models [10] as well as antioxidant properties [11]. Despite numerous reports of beneficial effects, however, other studies find deleterious consequences for MC treatment in some models of neurodegenerative disease and neuronal injury [12, 13] and damage to isolated mitochondria in micromolar concentrations [14]. Moreover, no single mode of action nor direct target of this antibiotic has been identified, which may partially account for the diversity of reported effects [15]. Genetic background is another feature that may contribute to these seemingly contradictory responses, as genetic variation amongst individuals is known to modify the functional activity of drugs [16]. Such gene-drug interactions are cumbersome to investigate in humans or even in vertebrate model organisms. Invertebrate genetic models offer a simplified means of investigating three-way interactions between genes relevant to disease susceptibility and progression, environmental conditions, and therapeutic agents.

 The genetic model organism, *Drosophila melanogaster*, is increasingly recognized as a useful model for neurodegenerative diseases due to its relative simplicity, ease of manipulation, high degree of conservation of neural mechanisms and signaling pathways, and availability of powerful genetic and molecular reagents for investigations of disease mechanisms [17, 18]. Thus, this model system should facilitate dissection of relevant response pathways during disease progression and identification of those alter responses to drugs such as MC.

 The effects of MC have been investigated mainly in mammalian models, although it has been shown that MC enhances survival of paraquat- (PQ-) fed *Drosophila* [19] and delays dopamine loss in a *DJ-1* genetic PD model in *Drosophila *[20]. We have developed a *Drosophila* model based upon ingestion of PQ, which recapitulates characteristic symptoms of PD with degeneration of dopaminergic neurons and accompanying neurological symptoms, including resting tremors and postural instability. Moreover, we demonstrated that mutations directly altering the regulation of DA homeostasis dramatically alter susceptibility to PQ [21, 22]. Thus, this model offers the ability to simultaneously modify genetic background and environmental toxins.

 In this paper, we confirm the results of previous studies [19, 20] utilizing this environmental toxin model to demonstrate that MC prolongs survival of PQ-exposed adult *Drosophila *and that it diminishes PQ-induced mobility defects, blocks associated changes in the DA homeostasis pathway, diminishes levels of reactive oxygen species (ROS), and prolongs DA neuron survival, as reported in the *Drosophila *DJ-1 model [20]. Moreover, we demonstrate that MC dose can dramatically alter the outcome of survival studies. We then extend the analysis of MC action by demonstrating that mutations in genes altering DA homeostasis and PQ susceptibility can affect the ameliorative action of MC. We further employ this system to identify signaling pathways that can modify PQ-induced toxicity and the protective effects of MC in this *in vivo Drosophila *model. Using loss of function mutants and overexpression transgenic lines, we found that JNK and Akt1 play an important role in protecting DA neurons against PQ toxicity and reductions in expression of these kinases diminishes the ability of MC to protect against PQ.

## 2. Materials and Methods

*Drosophila Strains and Culture Maintenance.* Two strains were utilized as wild type control lines in all experiments testing mutant strains: Canton S, a wild type strain, and *y* *w* ^1118^, a yellow-body, white-eye strain that is otherwise wild type. For all experiments employing Gal4 expression to drive UAS-transgenes, TH-Gal4/+, and UAS-transgene/+ were utilized as controls. Mutant strains obtained from the Bloomington *Drosophila* Stock Center were as follows: *rolled *(*rl* ^1^), a weak loss-of-function allele of the gene encoding ERK, *bsk* ^1^/*CyO* and *bsk* ^2^/*CyO* and *Df(2L)J27,* *bsk* ^[*J*27]^/*CyO*, *P*{*ry*[+*t*7.2] = *sevRas*1.*V*12}*FK*1 loss-of-function alleles of the gene encoding JNK, *ry* ^506^ *P*{*PZ*}*Akt*1^04226^/*TM*3, *ry* ^*RK*^ *Sb* ^1^ *Ser* ^1^ and *y* ^1^ *w* ^67*c*23^; *P*{*w* ^+*mC*^ *y* ^+*mDint*2^ = *EPgy*2}*Akt*1^*EY*10012^/*TM*3,  *Sb* ^1^ *Ser* ^1^ carrying loss-of-function alleles of *Akt/PKB*, a loss-of-function allele for the gene encoding reaper, *reaper*, *y* ^1^ *w* ^67*c*23^; *P*{*SUPor*-*P*}*KG*07184  *ry* ^506^, and a null mutant allele of *Nedd2-like caspase *(*Nc/Dronc*), *y* ^1^ w*; *Nc* ^51^/*TM*3,*Sb* ^1^. Transgenic strains employed for wild type expression of kinases were JNK (*bsk*), *y* ^1^ *w* ^1118^ *; P*{*UAS*-*bsk*.*A*-*Y*}*1* and *Akt1*, *y* ^1^ *w* ^1118^; *P*{*UAS*-*Akt*1}/*CyO*.

 The transgenic strain *UAS-2X eGFP *(Chromosome II) was obtained from the Bloomington *Drosophila* Stock Center and a *TH-Gal4* strain [23] was obtained from Jay Hirsh (University of Virginia). Loss-of-function *Catsup *alleles are described in Stathakis et al. [24], *pale* ^2^/*TM*3,  *Sb *is a loss-of-function mutation in the gene encoding TH [25]. The loss of function *Pu* ^*Z*22^ is described in Mackay et al. [26].  All mutants and transgenic strains were mated to the appropriate wild type strain, and all assays were performed on mutants heterozygous for the wild type genes. All stocks were maintained at 25°C.

### 2.1. Feeding Experiments

Separated male and female flies, 48–96 hr after-eclosion, were fed on filter paper saturated with one of the following solutions: 5% sucrose, 5% sucrose with 1 or 10 mM paraquat, minocycline HCl at varying concentrations, 10 mM PQ with 1 mM MC, and 1 mM PQ with 200 *μ*M NG-nitro-L-arginine methylester (L-NAME). Feedings were continued as indicated in Section 3. All chemicals were obtained from Sigma (St. Louis, MO).

### 2.2. Locomotion Assay

The mobility of adult male and female flies from each treatment group was assessed using a negative geotaxis climbing assay. A single fly was placed in an empty plastic vial, tapped to the bottom, and the time required to climb 5 cm was recorded three times sequentially with 10 min rest periods between each measurement. Each replication value recorded was an average of the three trials; each assay was conducted on 10 flies per test group.

### 2.3. HPLC Analysis

Monoamine and pteridine levels were determined using an ESA CoulArray Model 5600A high performance liquid chromatography system. Fifty adult heads were extracted in 60 *μ*L 0.1 M perchloric acid, followed by centrifugation. Ten *μ*L aliquots of each extract were injected. Analyses were conducted on three replicas of each test set. Amines and pteridines were separated on a Phenomenex Synergi 4 *μ*m Hydro-RP column (4.5 × 150 mm) according to the method of McClung and Hirsh [27]. Separations were performed with isocratic flow at 1 mL/min. Amines were detected with the ESA CoulArray electrochemical analytical cell, Model 5011 (channel 1 at −50 mV, channel 2 at 300 mV). Pteridines were detected with a Linear Model LC305 fluorescence detector (excitation wavelength 360 nm and emission wavelength 456 nm). Analysis was performed using ESA CoulArray software.

### 2.4. GTPCH Assay

GTPCH activity was assayed as previously described [21]. Briefly, extracts were prepared from 30 heads of 3–5 days old adult males in 100 *μ*L 50 mM Tris, 2.5 mM EDTA, and pH 8.0. Extracts were centrifuged at 10,000 rpm for 10 min and the protein concentrations of supernatants were determined using the BioRad Protein Assay Reagent. GTP was added to extract corresponding to 45 *μ*g of protein to a final concentration of 0.2 mM in a final volume of 70 *μ*L. The mixture was incubated for 1 hr at 37°C to convert GTP to dihydroneopterin triphosphate (dNP3), followed by its oxidation in 30 *μ*L of 1% iodine and 2% potassium iodide in 1 M HCl and dephosphorylation with 2 units of alkaline phosphatase (Roche). Neopterin peaks were detected by fluorescence at excitation wavelength of 353 nm and emission wavelength of 438 nm.

### 2.5. Confocal Microscopy

Whole mounts of dissected brains from *TH-Gal4*; *UAS-eGFP*, *TH-Gal4*; *UAS*-*eGFP*/*UAS*-*bsk* ^*WT*^, and *TH-Gal4*; *UAS*-*eGFP*/*UAS*-*Akt* ^*WT*^ adults fed with sucrose alone, with paraquat, or with paraquat and minocycline together were examined for dopaminergic neuron morphology and number, detected by visualizing GFP-expressing neurons. Each brain was scanned to include 15–18 sections for optimum visualization of DA neurons. The Z-sections were then utilized to get the average of all sections using a Leica TCS SP2 AOBS confocal microscope except for confocal images in Figure 8, which were captured using Zeiss LSM 710 Confocal Microscopy.

### 2.6. Catalase Assay

Crude enzyme extracts from adult flies fed PQ and MC as described above were prepared from ten heads from each treatment group in 150 *μ*L 0.1 M sodium-potassium phosphate buffer containing 0.1 M Triton X-100 (pH 7.0), and activity assays were conducted following the method of [28]. The reaction of head extract with H_2_O_2_ was determined at absorbance wavelength 230 nm and calculated using a molar extinction coefficient for H_2_O_2_ of 62.4. One unit of catalase activity was defined as 1 *μ*mole of H_2_O_2_ decomposed per min. All values represent the average of 6–8 replications from independently prepared extracts.

### 2.7. Lipid Peroxidation Assay

Head extracts from fifty heads were prepared in 100 *μ*L of 0.1 M phosphate buffer from female flies at 2–4 days after eclosion fed as described above for 24 hr. Two mL of reagent TCA-TBA (thiobarbituric acid)-HCl was added to 1 mL of head extract and heated for 15 min in a boiling water bath to allow malondialdehyde, the product of the lipid peroxidase reaction, to develop a red chromophore, detected spectrophotometrically at 535 nm, as described by [29].

### 2.8. Griess Assay for NOS Activity

Head extracts from fifty heads were prepared in 100 *μ*L of 0.1 M phosphate buffer with 1 M KCl (pH 7.4). Following centrifugation to remove debris, the supernatants were mixed with freshly prepared Modified Griess reagent (Sigma) in a volume ratio of 1 : 1. After a 15 min incubation period at room temperature in dark, nitrite levels were measured spectrophotometrically at 595 nm, with concentrations of nitrite calculated against a silver nitrite-derived standard curve and data was presented as a concentration of nitrite generated by extracts of 50 fly heads.

### 2.9. Statistical Analysis

One-way ANOVA with Dunnett's post-test or by two-tailed Student's *t*-test were used to analyze the data using GraphPad Prism (San Diego, CA). The figure legends describe the analyses of the data.

## 3. Results

### 3.1. Effect of Minocycline on Paraquat-Induced Truncation of Life-Span

**Figure 1:**
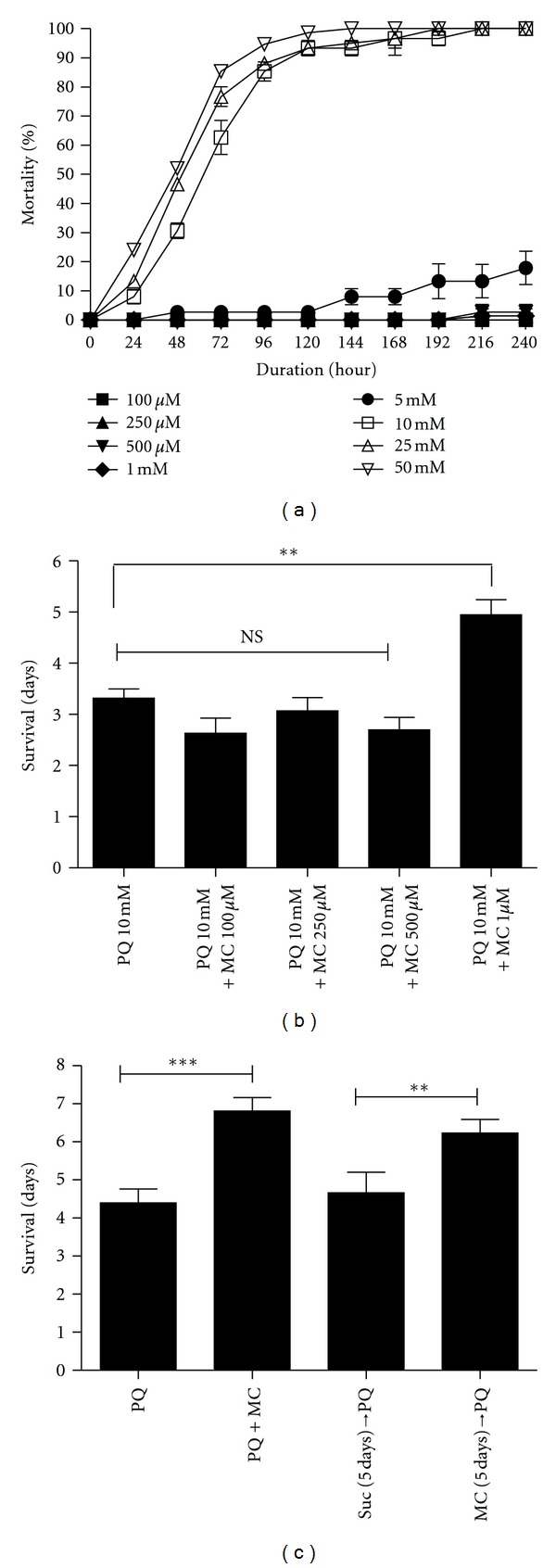
Effect of MC in absence and presence of 10 mM PQ on survival of adult male flies. (a) Effect of increasing concentrations of MC (100 *μ*M–50 mM) on survival of adult male flies. Data were collected every 24 hrs for each group until 100% mortality was noted with 50 mM MC. (b) Effect of different doses of MC co-fed with 10 mM PQ on the life span of wild type adult males. Co-feeding of 1 mM MC with 10 mM PQ significantly extends the survival duration compared with PQ alone. ** represents the difference between PQ-fed flies and those fed PQ with 1 mM MC at *P* < 0.005. NS = nonsignificant. Error bars indicate the standard error of the mean. Each data point was derived at least 10 replications of 15 flies each. (c) Wild type flies at 48 hrs after eclosion were fed PQ and/or MC using different regimens as shown in the graph. The MC co-feeding group was compared to the PQ alone group and the MC prefeeding group was compared with the same aged group prefed 5% sucrose. Both co-feeding and prefeeding regimens improved the survival duration compared to PQ alone. ** = *P* < 0.005 and *** = *P* < 0.001 and represent the significant difference between PQ and prefeeding and co-feeding groups. Error bars represent SEM, and *n* = 50–60 flies for all groups in (a), (b), and (c).

Because MC toxicity has been reported in some mammalian disease models, we first tested a range of MC concentrations from 100 *μ*M to 50 mM on non-PQ-treated flies to assess potential deleterious effects. We found that ingestion of 5 mM MC or greater affected viability, while lower concentrations caused no observable toxicity (Figure 1(a)). The results of this test for adult males are shown in Figure 1(a); females gave comparable results (data not shown). All subsequent experiments utilized MC concentrations of 1 mM or less to avoid drug-related toxicity.

 We then asked whether concentrations of antibiotic at 1 mM or lower could rescue the toxic effects of 10 mM PQ (Figure 1(b)). When exposed to PQ alone, the average survival duration of adult males was approximately three days. Cofeeding of MC at concentrations of 500 *μ*M and below did not improve survival duration; however, co-feeding of 1 mM MC with PQ extended the average survival duration an additional 48 hr, from three to five days (Figures 1(b) and 1(c)). Unless otherwise noted, all subsequent experiments were performed using 10 mM PQ and 1 mM MC. We then tested the efficacy of MC under three different regimens, comparing PQ and MC co-feeding, prefeeding MC for 2 days prior to PQ exposure, and prefeeding PQ for 2 days prior to exposure to MC alone. We found that neither prefeeding nor posttreatment of MC were able to modify the survival duration of PQ-exposed flies (data not shown). However, extending the MC pre-feeding period to 5 days resulted in extension of life span to almost the same degree as the co-feeding regimen (Figure 1(c)).

### 3.2. MC Protects against PQ-Induced Mobility Defects

**Figure 2:**
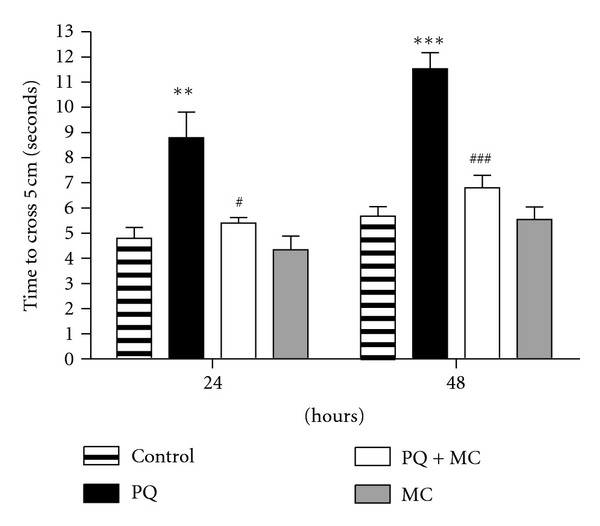
MC protects against PQ-induced mobility defects. The time required for adult male flies to climb 5 cm, after 24 and 48 hr of exposure to 10 mM PQ or 10 mM PQ with 1 mM MC flies was assayed. The ingestion of 1 mM MC alone has no effect on mobility, while ingestion of PQ alone adversely affects mobility. PQ and MC co-feeding results in mobility performance near control levels. The * and ^#^ indicate the significance of differences between control and PQ-fed flies and between PQ only and co-fed flies, respectively. ***/^###^ indicate *P* < 0.001,** indicates *P* < 0.005, and ^#^ = *P* < 0.05. Error bars represent standard error of the mean. Each data point represents at least 15 replications of 10 flies each.

We employed a climbing assay, which is a sensitive indicator of the onset of dopaminergic neuron-linked movement dysfunction, to assess whether MC could rescue the mobility deficits induced by PQ, similar to those observed by Faust et al. [20], in a *Drosophila DJ-1* genetic model of PD and in several mammalian models [30, 31]. Within 24 hr of the initiation of PQ feeding in the absence of MC, tremors and bradykinesia were apparent. At 48 hr, these flies were unable to climb and exhibited a strong bradykinesia-like behavior. In contrast, when PQ was cofed with MC, no movement defects were apparent, and mobility was comparable to the negative geotaxis activity of control and minocycline-only flies (Figure 2).

### 3.3. MC Delays PQ-Induced Loss of Dopaminergic Neurons

**Figure 3:**
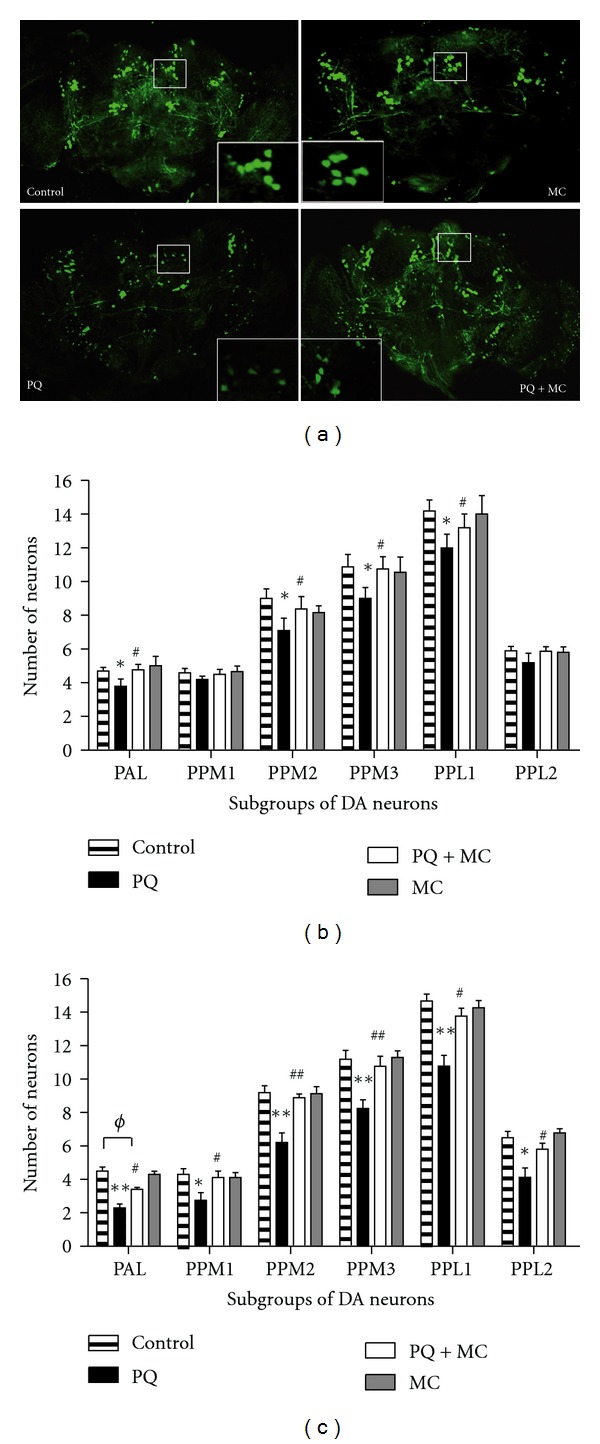
MC confers protection to dopaminergic neurons. (a) The effect of a 24 hr exposure of adult males to sucrose (Control), 1 mM MC alone (MC), 10 mM PQ alone (PQ), and 10 mM PQ with 1 mM MC (PQ + MC) on the dopaminergic neurons of *TH-GAL4; UAS-eGFP* adult brains. The inset in each image demonstrates the change in the morphology and number of the PPM2 subgroup of neurons. The exposure to MC alone (MC) and sucrose (control) does not alter the number or morphology of the dopaminergic neurons. The addition of MC to PQ delays neuron loss and onset of abnormal neuron morphology relative to PQ only. Scale bar for whole brain images = 100 *μ*m. (b) and (c) MC delays PQ-induced selective loss of dopaminergic neurons. The average number of neurons per subset was determined 24 hr (b) and 48 hr (c) after the initiation of feeding and shows that MC delays, but does not prevent, PQ-mediated neuronal loss in different dopaminergic neurons. Each subset of dopaminergic neurons was scored separately in 15–25 brains. The significance of the difference in each neuron cluster between the PQ-treated and control groups and between the PQ-treated and co-fed groups is indicated as * and ^#^, respectively, where * = *P* < 0.05, ** = *P* < 0.005, ^#^ = *P* < 0.05, and ^##^ = *P* < 0.005. ^*ϕ*^ represents the significant difference between control and PQ with MC co-fed brains where ^*ϕ*^ = *P* < 0.05. Error bars represent standard error of the mean.

We previously established that the onset of movement dysfunction upon PQ ingestion coincides with the loss of region-specific subsets of dopaminergic neurons in the adult brain [21]. In light of the ability of MC to ameliorate PQ-induced tremors and mobility deficits, we next asked whether this effect is mediated through protection of at-risk dopaminergic neurons. Dopaminergic neurons were detected by the TH promoter-directed expression of GFP in the transgenic strain, *TH-Gal4; UAS-eGFP*. We compared dopaminergic neuron morphology and numbers in brains at 24 and 48 hr after the initiation of feeding 5% sucrose only, PQ only, MC only, or PQ with MC (Figure 3).  Figure 3(a) displays characteristic results in dopaminergic neuron cluster PPM2 after 24 hr of treatment. The number and morphology of these neurons after the MC-only treatment were indistinguishable from neurons in control brains. As we had observed previously, using both the GFP reporter and immunolocalization of TH to identify dopaminergic neurons [21], these neurons display characteristic patterns of morphological changes and neuronal sensitivity after PQ exposure (Figure 3(a)), whereas neurons in animals exposed to MC with PQ retained near normal morphology and neuron number (Figures 3(a), 3(b), and 3(c)). Upon treatment with PQ only (Figure 3(a)) for 24 hrs, we observed that the PAL subgroup in the anterior region and the PPL1, PPM2, and PPM3 subgroups in the posterior brain exhibited statistically significant neuron loss relative to controls (Figure 3(b)). In the animals that were co-fed PQ and MC, there was no neuron loss in the PPM1 or PPL2 subgroups at 24 hr, and neuron numbers and morphology in other clusters were almost identical to those in control brains (Figures 3(a) and 3(b)). After 48 hrs of PQ feeding, previously affected clusters continued to deteriorate, while neurodegeneration was noted in the previously unaffected PPM1 and PPL2 clusters, while improved survival of all dopaminergic neuron groups was noted in the PQ and MC co-fed flies (Figure 3(c)). Therefore, we conclude that the extension of life span observed when flies were fed MC along with PQ and delay of the onset of movement deficits correlate with protection against PQ-induced neurodegeneration.

### 3.4. MC Blocks Changes in DA Pathway Components Indicative of PQ-Induced Oxidative Stress

**Figure 4:**
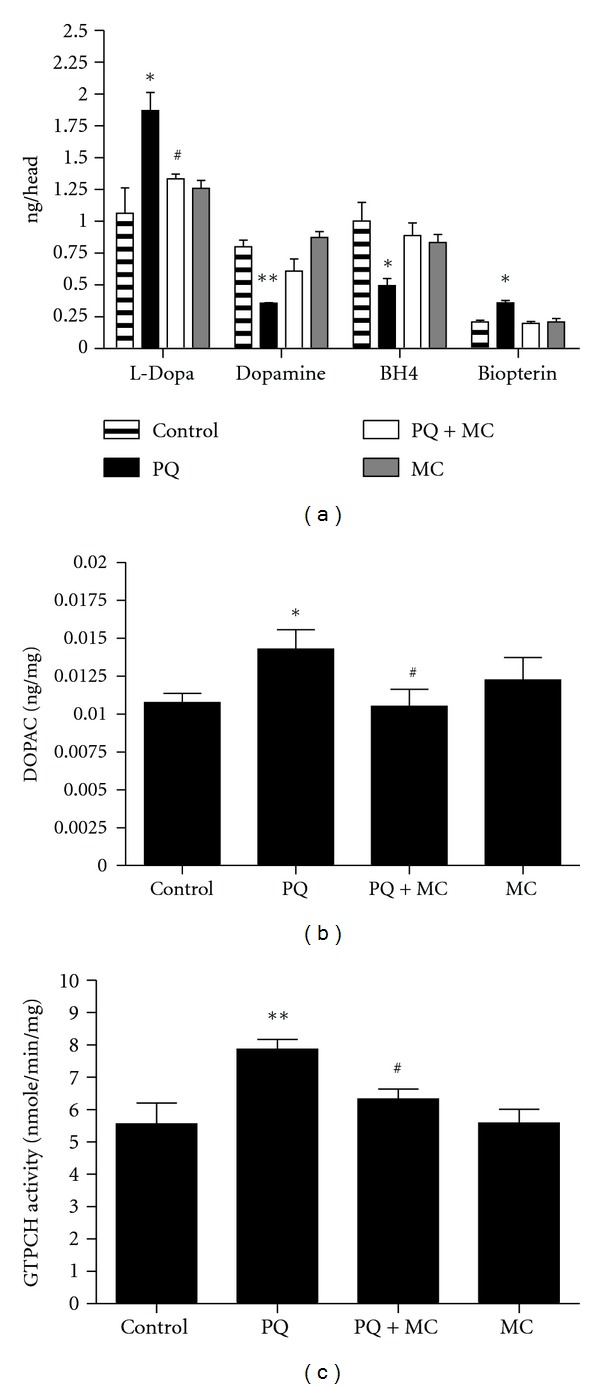
MC blocks the changes induced by PQ in the DA and BH_4_ biosynthesis pathways. (a) Changes in the DA and BH_4_ metabolites in adult males exposed to 10 mM PQ or 10 mM PQ with 1 mM MC for 24 hrs. The increase in L-DOPA levels, indicative of PQ-stimulated TH activity, is reduced by MC. (b) The DA metabolite, DOPAC, is elevated by PQ exposure and is significantly decreased by the co-feeding of MC to male adults for 24 hr. (c) The compensatory increase in GTPCH activity in PQ-fed adult males is reduced in PQ-MC co-fed males at 24 hr of ingestion. The significance of differences in each subset between the PQ-treated and control groups, and PQ-treated and co-fed groups is indicated as * and ^#^, respectively, where * and ^#^ = *P* < 0.05 and ** and ^##^ = *P* < 0.005. Error bars represent the standard error of the mean. Each data point represents at least 10 replications of 15 flies each.

The production of DA is rate-limited by two enzymes: tyrosine hydroxylase (TH), which converts tyrosine to L-DOPA, and GTP cyclohydrolase (GTPCH), which is rate-limiting for the production of tetrahydrobiopterin (BH_4_), a cofactor for and regulator of TH catalysis. We previously observed that sensitivity to PQ is at least partially defined by the activity of the BH_4_ and DA biosynthesis pathways [21]. We found that following PQ ingestion, but prior to loss of dopaminergic neurons, there is a transient stimulation of DA pathway activity, followed by a decrease in BH_4_ and DA levels and a corresponding increase in oxidative products, biopterin and DOPAC, respectively. The observed protection by minocycline against the effects of PQ might be mediated through interactions with the DA homeostasis machinery, either modulating DA synthesis or transport or through its ability to act as a scavenger of ROS [11], delaying the PQ-induced oxidative depletion of DA and subsequent oxidative damage. Ingestion of MC alone for 24 hr has no significant effect on the production of pathway metabolites or on GTPCH activity (Figure 4), ruling out the possibility that MC modulates DA homeostasis. PQ induced dynamic changes of enzyme activity, pathway products, and oxidative products. After 24 hr of PQ exposure, L-DOPA pools are significantly elevated, indicating an increase in TH activity; however, DA was depleted and the DA metabolite, DOPAC (3,4-dihydroxyphenylacetic acid) was elevated, as expected in an oxidative environment (Figures 4(a) and 4(b)). Similarly, GTPCH activity increased (Figure 4(c)), while BH_4_ pools are diminished and the oxidized product biopterin increased with PQ exposure (Figure 4(a)). In contrast, when MC was co-fed with PQ for 24 hr, the effect of PQ on each of these components was significantly less severe, consistent with the reported antioxidant property of MC.

### 3.5. MC Reduces PQ-Generated Reactive Oxygen Species

**Figure 5:**
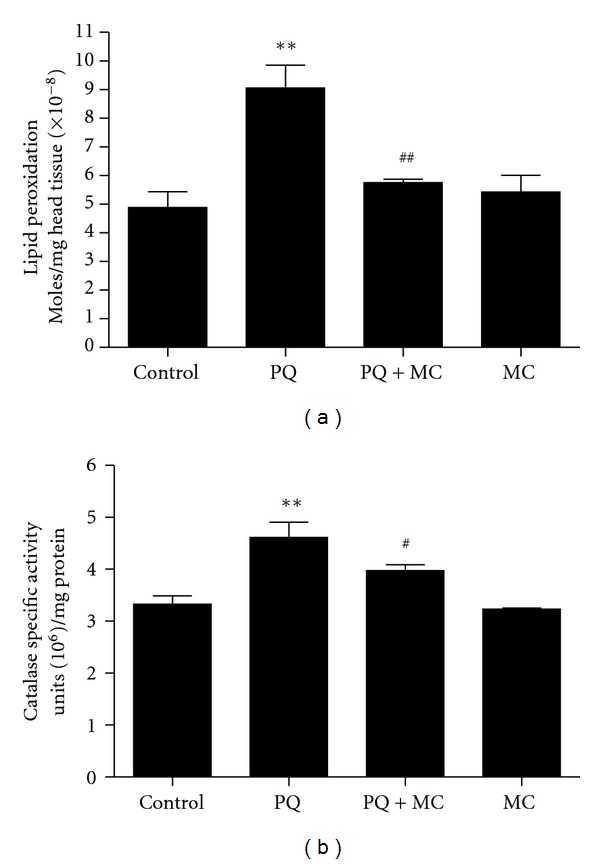
MC reduces PQ-generated reactive oxygen species. (a) The level of lipid peroxidation product was determined in head extracts of males that had ingested PQ or PQ with MC for 24 hr. MC significantly decreases the amount of lipid peroxidation induced by PQ. (b) MC reduces the specific activity of catalase after 24 hr of ingestion in co-fed male flies. The significance of differences between the PQ-treated and control groups and between the PQ-treated and co-fed groups was indicated as * and ^#^, respectively, where * and ^#^ = *P* < 0.05. Error bars represent standard error of the mean. The experiments were done as 5–8 replicas of 10 head extracts.

The ability of MC to block the PQ-induced changes in the DA and BH_4_ biosynthesis pathways indicative of oxidative stress led us to hypothesize that MC was serving principally as a scavenger of PQ-generated ROS in *Drosophila*. We employed two assays for oxidative stress, lipid peroxidation [29], and induction of catalase activity [28, 32]. PQ ingestion results in a two-fold elevation of lipid peroxidation within the first 24 hr (Figure 5(a)). Co-feeding of MC with PQ almost completely blocks this indicator of lipid damage. Elevated catalase activities were detected in the heads of both PQ and PQ plus MC groups (Figure 5(b)); however, the catalase activity in flies that had ingested MCwith PQ was significantly lower than those exposed to PQ only. These results strongly suggest that MC has a strong capacity to suppress ROS generated by PQ.

### 3.6. MC Cannot Rescue *Pu* (GTP Cyclohydrolase) Mutants

**Figure 6:**
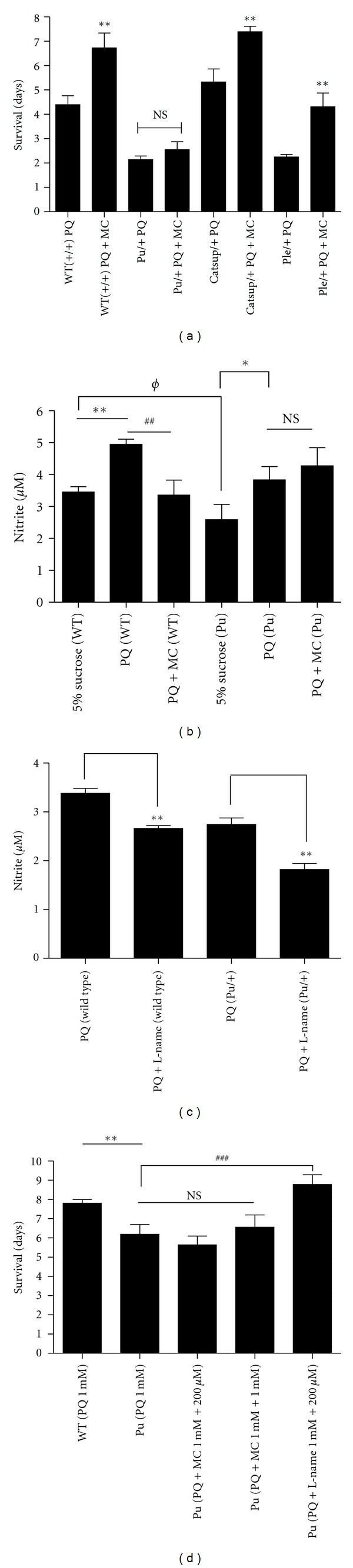
MC fails to rescue *Punch *mutants due to dysregulation of nitric oxide synthase catalytic function. (a) Effect of 1 mM MC co-fed with 10 mM PQ on DA regulatory mutants, *Catsup* ^26^/+, *Pu* ^*Z*22^/+ and *ple* ^2^/+. *Catsup *and *ple *mutant flies showed extension of life span, while *Pu* mutants did not. NS = not significant. ** = *P* < 0.005 and represents significant differences between PQ and PQ with MC. (b) After 24 hr of PQ, or PQ with MC exposure, suppression of NOS was detected in wild type heads but not in *Punch *mutants where NO levels of non-PQ-treated *Pu* mutants assayed are significantly lower than NO levels of non-PQ-treated wild type heads. * or ^*ϕ*^ *P* = <0.05 and ** or ^##^ *P* = <0.005. (c) Co-feeding of L-NAME with 1 mM PQ reduced NO production in wild type and *Pu* mutants. ** = *P* < 0.005. (d) The survival of *Pu *mutants was improved by co-feeding PQ with L-NAME, but not with MC when compared with survival of *Pu *mutants on PQ alone. * represents significant differences between control and PQ-exposed flies, while ^#^ represents significant differences between flies fed PQ only and PQ with MC. ** = *P* < 0.005 and ^###^ = *P* < 0.001. NS = not significant. Error bars represent standard error of the mean and *n* = 100–120 fly heads for NOS assays and 50–60 flies for survival where experiments were replicated thrice with the same sample size.

Mutations in the rate-limiting genes for BH_4_ and DA synthesis, *Punch* (*Pu*; GTPCH) [33] and *pale *(*ple*; TH) [25], respectively, result in decreased DA pools in adult heads and increased sensitivity to PQ [21]. Conversely, loss-of-function alleles of *Catecholamines up* (*Catsup*), a negative regulator of TH and GTPCH, have elevated BH_4_ and DA pools and a strong resistance to PQ [22, 24]. While we continue exploring the mechanistic basis for the differential sensitivity to PQ, these mutant strains provided the opportunity to begin defining genetic components that might have roles in modulating the protective effects of MC. We found that *Catsup* heterozygotes survive PQ 24 hrs longer, on average, than wild type adults, while *Pu* and *ple* heterozygotes die 48 hrs or more before wild type flies (Figure 6(a)). MC extended the survival of wild type flies and the *Catsup* and *ple* mutants, by approximately 2 days for each strain. Therefore, we detected no DA-specific interactions with MC in these mutants.

Strikingly, however, MC was unable to improve the survival of *Pu* mutants under these conditions. One possibility is that the heterozygous *Pu* mutants might provide a sensitized background which reveals an otherwise undetectable deleterious effect of 1 mM MC. However, in other studies, we found that ingestion of MC at concentrations up to 5 mM by *Pu *mutants had no discernable effect on survival (Ajjuri and O'Donnell, in preparation). Alternatively, oxidative damage in *Pu* mutants, perhaps related to a *Pu* function other than regulation of DA synthesis, might progress too rapidly for MC to impart its protective effect. One such candidate is nitric oxide synthase (NOS), which requires BH_4_ as a cofactor. It is well known that limiting BH_4_, which should be a consequence of loss-of-function mutations in *Pu*, results in the catalytic uncoupling of NOS, dramatically enhancing oxidative stress through production of elevated peroxides and peroxynitrites [34, 35]. Since *Pu *mutants have reduced levels of BH_4_ [33], we hypothesized that the failure of MC protection in *Pu* mutants is linked to catastrophic oxidative damage stemming from induction of NOS by PQ and its subsequent uncoupling. If this hypothesis is correct, then inhibition of NOS catalytic activity should limit the production of ROS and RNS and, thereby, improve survival of the *Pu *mutant flies. As expected, heterozygous *Pu *mutants have lower NOS activity than wild type flies. Ongoing experiments to date have revealed no detectable effects of MC alone on NOS activity (unpublished data). PQ ingestion resulted in elevated NO production, as determined by the Griess assay, in both wild type and *Pu *mutant heads (Figure 6(b)). MC reduced nitrite levels in wild type flies, suggesting its ability to reduce the inflammatory response in flies as in mammals. However, MC was ineffective in reducing nitrite levels in *Pu *mutants, exposed to 10 mM PQ (Figure 6(b)). We then fed 1 mM PQ, to slow the rate of accumulation of oxidative damage, in an effort to further dissect events contributing to neurodegeneration. We observed that NOS inhibitor L-NAME reduced nitrite levels in both wild type and *Pu *mutants (Figure 6(c)). Moreover, ingestion of L-NAME was able to prolong survival of the *Pu *mutant strain as well as wild type flies exposed to 1 mM PQ (Figure 6(d)). In contrast, even at this 10-fold lower concentration of PQ, MC was still unable to improve the survival of *Pu *mutants. These results suggest that the failure of MC to rescue *Pu *mutants was due to its inability to limit NOS activity and therefore reduce oxidative damage when BH_4_ production is compromised.

### 3.7. Loss of Function Mutants of the Genes Encoding *JNK* and *Akt* Are Sensitive to 1 mM PQ but Involvement of Reaper, Caspase, and Rolled in PQ-Induced Toxicity Was Not Detected

Signal transduction pathways associated with oxidative stress, inflammatory, and apoptotic responses are likely targets of minocycline action [15]. However, it also is known that dopaminergic signaling and homeostasis, highly conserved processes in flies and mammals, are highly responsive to environmental stressors across species lines [36, 37]. This feature of dopaminergic function is highlighted by our findings that PQ induces early changes in DA metabolism and that mutations in genes altering DA homeostasis strongly affect the sensitivity of *Drosophila* to PQ [21, 22]. The roles of such signal transduction pathways are likely to be highly complex, as discrepancies in evidence for participation of particular kinases in responses to oxidative damage and subsequent neurodegeneration emphasize [38, 39]. As a foundation towards employing our whole organism disease model to better understand the interface between regulation of signal transduction and dopamine homeostasis in dopaminergic neurodegeneration, we next turned our attention to MC effects under conditions in which specific signaling pathways previously implicated in neurodegeneration mechanisms are genetically modified.

 The mitogen-activated protein kinase (MAPKs) subfamilies, extracellular signal regulated kinases (ERK), c-Jun N-terminal kinases (SAPK/JNK), and p38 kinases, are known to be activated by a wide range of stimuli including inflammatory cytokines and diverse environmental stressors and to mediate a variety of downstream effects likely to be integral to protection against neurodegenerative mechanisms [40, 41]. In mammalian models for neuronal injury and neurodegenerative diseases, pharmacological approaches have provided evidence that p38 and JNK are mainly implicated in neuronal death processes, while ERK may promote cellular recovery/survival from neuronal death implicated in these conditions [42].

We therefore tested heterozygous mutants for JNK, encoded by the gene *basket* and Akt/PKB, encoded by *Akt1*, and ERK, encoded by *rolled*, in *Drosophila*. In addition, we tested mutations in the caspase-9 gene, *dronc*, and mutations in the proapoptotic gene, *reaper*. These kinases function in myriad biological processes but are particularly known to respond to various cellular stress. We hypothesized that they should also play key roles in the toxic responses triggered by PQ.

**Figure 7:**
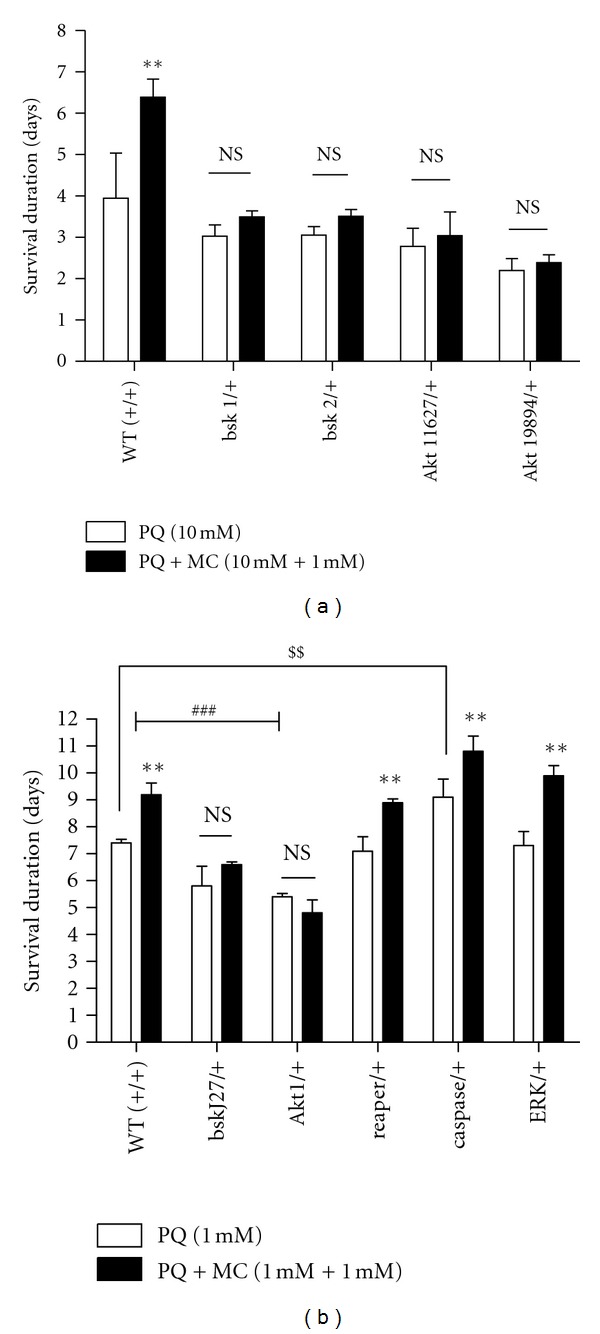
PQ and MC effects on viability are modified by components of stress response pathways. (a) Effect of *bsk *and *Akt *loss-of-function mutant alleles on PQ and MQ effects on survival. Treatment with 1 mM MC failed to improve the survival of* bsk* and *Akt* loss-of-function mutants fed 10 mM PQ. *n* = 120 and each data point represents at least three independent replications of 40 flies for each.genotype. (b)  Effect of 1 mM PQ and 1 mM MC on loss-of-function mutants of signaling pathways, JNK, Akt, reaper, caspase, and ERK. *rolled *(ERK), *reaper *and *caspase *mutants showed an extension of life span with MC treatment, while the survival of JNK and Akt mutants was unmodified in the presence of MC. ** = *P* < 0.005 represents significant difference between PQ and PQ with MC groups. ^##^ = *P* < 0.005 and shows a significant difference between PQ-fed wild type and PQ-fed *bsk *and* Akt* loss-of-function mutants. NS = not  significant. ^$$^ = *P* < 0.005 represents significant difference between PQ-fed wild type and PQ-fed caspase loss-of–function mutant. Error bars represent standard error of the mean. *n* = 180 and each data point represents at least three independent replications of 50–60 flies each.

 We exposed heterozygous mutants with loss of function alleles of *bsk* and *Akt *to 10 mM PQ alone or in combination with 1 mM MC and observed that both sets of mutants had apparently slightly elevated sensitivity to PQ although not statistically significant (Figure 7(a)). Interestingly, however, MC failed to improve the survival of either mutant, while survival of the control strain was significantly improved. To clarify the effects of mutations in these kinase genes, we tested additional *bsk* and *Akt *alleles, decreasing the PQ concentration (1 mM) to slow the progression of PQ-induced damage as in the *Punch *mutant experiments described above. In this experiment, we expanded our analysis to include a mutant allele of the ERK gene, *rolled*, and mutants for the apoptotic pathway genes *dronc* (Caspase) and *reaper*. Under these conditions, the heterozygous *bsk* and *Akt* mutants showed increased sensitivity to PQ, dying, on average, two days before wild type flies, and again MC was ineffective in rescuing these mutants (Figure 7(b)). These results suggest either that JNK and Akt signaling pathways are important in the protective response of MC in flies or that diminution of their activity heightens the oxidative environment to a point that the beneficial effects of MC are lost. However, the sensitivity of heterozygous *reaper *and *rolled *mutants to PQ and the ability of MC to increase survival were indistinguishable from the wild type controls. In contrast, the heterozygous caspase mutant, *dronc*, survived two days more than the wild type controls on PQ and minocycline improved survival an additional two days (Figure 7(b)).

### 3.8. Overexpression of *JNK* and *AKT* Confers Protection against Paraquat-Induced Toxicity in Dopaminergic Neurons

**Figure 8:**
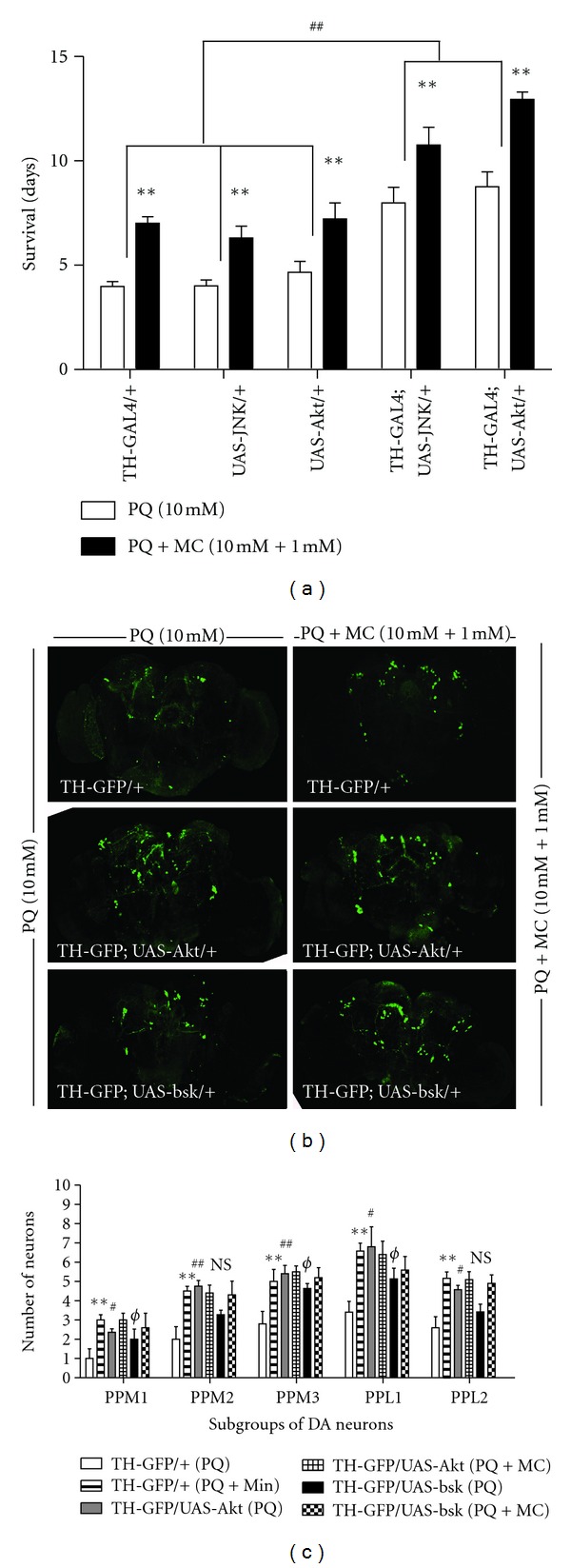
Overexpression of wild type BSK/JNK and Akt provides protection against PQ. (a) Adult males of genotypes *TH*-*GAL4*/+, *UAS*-*bsk* ^1^/+, *UAS*-*Akt* ^1^/+, *TH*-*GAL4*; *UAS*-*bsk* ^1^/+, and *TH*-*GAL4*; *UAS*-*Akt* ^1^/+ flies were fed, beginning at 48 hr after eclosion, 10 mM PQ or 10 mM PQ and 1 mM MC. The average survival duration for each group was determined. ** = *P* < 0.005 and represents significant differences between the PQ and PQ with MC groups. ^##^ = *P* < 0.005, indicating a significant difference between control and JNK or Akt expressing flies fed only PQ. Error bars represent standard error of the mean. Each data point represents at least three independent replications of 50–60 flies each. (b) The effect of PQ (10 mM) and PQ + MC (10 mM + 1 mM) on brains of transgenic lines shown in the images. The overexpression of wild type Akt and Bsk/JNK in dopaminergic neurons provides protection to DA neurons against PQ, however; addition of MC in fails to provide additional protective to transgenic lines, *TH-Gal4;* *UAS*-*eGFP*/*UAS*-*Akt* ^*WT*^ and* TH-GAL4;* *UAS*-*eGFP*/*UAS*-*bsk* ^*WT*^ against PQ. (c) The average number of neurons per subset was determined 24 hr after the initiation of feeding in these transgenic lines. MC ingestion significantly improved the survival of DA neurons against PQ-induced loss of DA neurons in *TH-Gal4; UAS-eGFP/+* brains.The overexpression of wild type Akt and JNK supports the survival of DA neurons against 10 mM PQ but addition of 1 mM MC failed to provide additional protection against PQ mediated DA neuron loss in these transgenic lines. ** = *P* < 0.005 and represent significant differences between PQ and PQ and MC-treated *TH-Gal4; UAS-eGFP/+* brains. ^#^ represents significant differences between PQ-treated *TH-Gal4; UAS-eGFP/+ *and PQ-treated *TH-Gal4;* *UAS*-*eGFP*/*UAS*-*Akt* ^*WT*^ and ^Φ^ represent significant differences between PQ-treated *TH-Gal4; UAS-eGFP/+* and PQ-treated *TH-GAL4;* *UAS*-*eGFP*/*UAS*-*bsk* ^*WT*^ brains, respectively. ^#/Φ^ = *P* < 0.05 while ^##^ = *P* < 0.005. *n* = 8–12 brains for each transgenic lines. NS = not significant.

The observations that *JNK* and *Akt* loss-of-function heterozygous mutants increase the sensitivity to PQ and that normal expression of these genes appears to be important for MC-mediated protective responses against PQ led us to test whether this effect is reversed when JNK and Akt are overexpressed. Since PQ toxicity initially is observed in dopaminergic neurons and DA itself appears to interact in this process, we drove expression of wild type JNK and Akt in DA neurons using the GAL4-UAS system [43]. Even though we employed PQ at the higher concentration of 10 mM for these experiments, the expression of JNK and Akt in dopaminergic neurons resulted in approximately a two-fold increase in the survival duration in both cases (Figure 8(a)). MC improved life span in all strains by 30% compared to 10 mM PQ alone. Importantly the combination of MC plus overexpression of Akt in dopaminergic neurons enhanced survival over three-fold relative to the wild type control. These results demonstrate that both Akt and JNK have prosurvival functions in PQ-induced DA toxicity, with Akt expression having the strongest effect.

We further verified the protective effect of wild type JNK and Akt by examining the survival of at-risk dopaminergic neurons in the adult brain. The transgenic strain, *TH-Gal4; UAS-eGFP* was crossed with *UAS*-*JNK* ^*WT*^and  *UAS*-*Akt* ^*WT*^, expression of GFP in concert with JNK or Akt. We observed that MC was able to protect against neuron loss in subgroups of posterior brain DA neurons expressing GFP, but otherwise wild type, relative to the brains of flies from the same cultures exposed to PQ only (Figure 8(b)). The overexpression of Akt or JNK in dopaminergic neurons prevents PQ-induced DA neuron loss in most subgroups of DA neurons relative to the control brains. Similarly, the transgenic brains, *TH-GAL4;* *UAS*-*eGFP*/*UAS*-*bsk* ^*WT*^ that were fed PQ (Figure 8(b)) displayed significantly greater DA neuron numbers. However, cofeeding of 1 mM MC with PQ failed to provide neuroprotection above that observed in Akt or JNK overexpression (Figures 8(b) and 8(c)). These results are in line of the survival data where the protective effect of MC on transgenic lines, *TH-GAL4;UAS-JNK *and *TH-GAL4;UAS-Akt/+*, was proportionate to those observed with control flies, rather than further enhancing survival.

## 4. Discussion

### 4.1. MC Imparts Antioxidant Effects in a PQ-Induced *Drosophila* Model for Parkinson's Disease

PQ is considered an oxidative stressor, generating superoxide and hydroxyl radicals [31]. Moreover, epidemiological and experimental studies point to PQ as an etiological agent for PD [44]. We have used PQ ingestion to establish an *in vivo Drosophila* PD model [21] and here employ this model to explore genetic interactions that alter the effects of MC, as a model for analysis of drugs offering therapeutic benefits in PD. As may be seen in the results reported in this study, this system has provided a fruitful avenue for exploring the effects of genetic alterations that may play roles in DA neuron susceptibility to deleterious environmental insult. It is noteworthy that we observe strong effects, both on PQ toxicity and on the protective capacity of MC, despite the fact that in all instances we are working with homozygous lethal mutant alleles, necessitating the use of heterozygotes with a wild type allele also present in each instance. It is increasingly recognized that dopaminergic neurons are at heightened oxidative risk, largely due to the interactive nature of DA itself. It is imperative that DA homeostatic mechanisms and oxidative surveillance are sensitively regulated and balanced to avoid catastrophic neuronal destruction. We note that comparable studies in mammalian models, incorporating a large collection of mutant alleles, are more difficult to implement. However, such studies are straightforward using the *Drosophila *model system, and our results with these heterozygous strains clearly support to prevailing view of the exquisite balance of pathways needed for the well-being of dopaminergic neurons.

MC has been shown to have anti-inflammatory and antioxidant properties in numerous neurodegenerative and injury-induced mammalian models [15, 45]. Although the exact biological targets for MC are still not well known, it has been reported that MC causes the inhibition of the cytochrome c release from mitochondria, the inhibition of caspase-1 and -3 expression, and the suppression of microglial activation [8, 15, 46].

Despite numerous reports supporting the efficacious role of MC in numerous models of neurological disease and injury, other studies have reported the absence of effects or even increased deleterious effects [12, 13]. Diguet et al. [12] reported that the protective or deleterious effects of MC depend on the mode of administration and dose of the drug. Because there is continuing controversy in MC studies, we first conducted a toxicity test employing a range of MC doses. Since we found that concentrations above 5 mM were toxic when fed to wild type adult flies, we employed 1 mM MC, a concentration substantially below toxic levels and yet effective in ameliorating the deleterious effects of 10 mM PQ. In addition, we tested various treatment paradigms and found that both pre-feeding and co-feeding regimens were equally protective. Bonilla et al. [19] also reported the prevention of PQ-induced reduction of survival duration in *Drosophila*, but the mechanism through which MC imparts this effect in *Drosophila *was not investigated in that study. More recently, Faust et al. [20] tested the efficacy of MC in ameliorating dopaminergic neuron loss in *Drosophila *when expression of DJ-1A was blocked by specific expression of *DJ-1A RNAi *in these neurons. In this study of a genetic model for PD, higher concentrations of MC (50–100 mM) were able to rescue the DJ-1RNAi-mediated loss of DA neurons. These investigators also assessed the effect of MC on DA pools and on the survival of DA neurons in the DCM region of the brain. Our study supports and extends this interesting study, demonstrating that this antibiotic is also effective in an environmental toxin model of PD. We also find improvement in survival and DA pools, but also a striking rescue of DA neurons, not only in the specific region investigated by Faust et al. but, interestingly, in all subsets of DA neurons. Mobility also was rescued in our study, as were markers of oxidative stress and inflammation. Thus, the efficacious effects of MC in both a genetic and a toxin model of PD in *Drosophila* produce comparable outcomes despite the fact that the *DJ-1* knockdown effects were observed at approximately 10 to 25 days after eclosion, while this PQ study tested more acute responses. Formally, there is a possibility that MC chemically interacts with and detoxifies PQ. However, the fact that comparable results are observed in both genetic and PQ models of PD in *Drosophila* argues strongly against this selective chemical interaction. Thus, we conclude that *Drosophila *systems present a robust model for investigating mechanisms of therapeutic action.

We have shown previously that PQ induces first a rapid activation of DA synthesis, as well as the BH_4_ cofactor pathway required for both DA biosynthesis and NOS activity, followed by rapid oxidative turnover of BH_4_ and DA [21]. We therefore expanded our neurochemical analysis to monitor L-DOPA and DOPAC. We detected suppression of each of these neurochemical responses upon MC treatment, confirming a strong antioxidant function for MC in this system from the perspective of DA metabolite responses as well as for the generation of ROS responses, by catalase and lipid peroxidation assays.

 Kraus et al. [11] compared the antioxidant property of MC with other known antioxidants. The antioxidant property was assessed on mammalian neuron cell culture via cell-based glutamate-induced oxidative stress assays and cell-free antioxidant activity assays including lipid peroxidation. MC along with other known antioxidants such as (±) tocopherol showed direct radical scavenging activity, proposed to be due to the presence of phenolic ring capable of reacting with free radicals, leading to the formation of relatively stable and unreactive phenol-derived free radicals [15]. Our results, combined with those of Bonilla et al. [19] and Faust et al. [20], strongly support a similar ability of MC in these whole organism studies under a variety of oxidative stress paradigms.

### 4.2. Failure of MC Protection against PQ in Punch Mutants

Having previously shown that mutants with defects in DA biosynthesis pathways show differential susceptibility to PQ [21], we employed these mutants to test for gene-environment interactions that might modify the efficacy of MC. Interestingly, MC improved the survival of *pale *and *Catsup *mutants proportionate to those with wild type flies, while it failed to rescue *Pu *mutants. While one possible explanation for this lack of effect is that a deleterious MC effect is revealed in the *Pu *mutant, we have observed no evidence of altered survival when MC alone, at concentrations employed in this study, is ingested by this mutant strain. These results suggested that functions of *Pu *other than its role in DA homeostasis might impact its effect on MC protection. BH_4_, the terminal product of the GTPCH pathway, also functions as an essential cofactor for NO production, facilitating the dimerization of NOS and functioning as a single electron donor during the process of NO production [35]. The limiting BH_4_ pools, as in heterozygous *Pu *mutants, cause the uncoupling of electron transfer in NOS catalytic function, in consequence, the generation of superoxide and peroxynitrites radicals [35, 47]. In support of this interpretation of our results, we found that *Punch *mutants exhibited lower than normal NOS activity and that *in vivo *inhibition of NOS catalytic function with the inhibitor L-NAME improved the survival of PQ-fed flies. The inability of MC to prevent excessive generation of peroxynitrites in *Punch *mutants presents an example of modified drug-gene interaction and may help to explain the phenomenon of drug failure response in PD and other neurodegenerative diseases. Our data also validates *Drosophila *as an *in vivo *model for screening of drug molecules for possible drug-gene interaction.

### 4.3. Identification of Signal Transduction Pathways Necessary for MC Protection against PQ-Induced Neurotoxic and Neuroinflammatory Responses

Our investigation of signaling pathways mediating PQ-induced neurotoxic in *Drosophila* seeks insights into signal transduction pathways that can modify PQ-mediated toxicity at the whole organism level. Most of the previous mammalian studies have utilized *in vitro *(i.e., cell culture) approaches to address this question. Moreover, these mammalian studies have used primarily pharmacological inhibitors to block the proposed functions of the signaling pathway, which may lack complete specificity of function. Finally, the variations in the type of inhibitor used, inhibitor concentrations, and time of addition as well as cell lines may affect the outcome of these experiments [48, 49].

 We have tested the roles of kinases and proapoptotic genes known to be functionally conserved with mammals. The heterozygous loss-of-function mutants for *JNK/bsk* (*bsk* ^*J*27^, *bsk* ^1^ and *bsk* ^2^) and *Akt *(*Akt* ^1^, *Akt* ^11627^ and *Akt* ^19894^) exhibit sensitivity to PQ in the absence of MC and fail to respond to antibiotic treatment. Moreover, overexpression of the wild type form of either kinase results in resistance to PQ. These results indicate crucial roles for these signaling pathways against neuronal stress responses to PQ. In contrast, heterozygous loss-of-function mutants for ERK and reaper mutants were equivalent to wild type flies in their sensitivity to PQ. We note that this lack of effects does not necessarily indicate an absence of roles for these latter genes. Leaky mutations or redundant functions could easily prevent the detection of deleterious effects. Ongoing studies will address these issues more fully.

In mammals, Akt1 plays a crucial role in cell survival and also is regulated by the PI3K-mediated signaling pathway [41]. Deregulation of the Akt1-mediated signaling pathway has been well documented in familial and sporadic forms of PD models [38, 50]. Stimulation of the Akt1 signaling pathway in *in vitro *or *in vivo *models resulted in neurotrophic, antiapoptotic effects [41]. Yang et al. [50] found suppression of ROS and survival of DA neurons in transgenic strains over-expressing *Drosophila *Akt1 in DJ1 RNAi strains, again illustrating that the PQ model parallels genetic PD models in many respects.

 In mammalian PD models, JNK, which initiates programmed cell death by inactivating the antiapoptotic protein Bcl-xl, is activated in TH neurons in a PQ-induced mammalian PD model suggesting a role JNK in neurodegeneration [51]. Similarly, activated JNK has been detected in Parkin mutants in *Drosophila *[52]. SP600125, a specific JNK inhibitor cofed with 20 mM PQ increased the survival and locomotory activity when compared with those fed with 20 mM PQ [53]. Moreover, Wang et al. [54] also demonstrated important roles for JNK in longevity and resistance to PQ-induced oxidative stress. Our data suggest a specific role of JNK in the survival response of DA neurons in our PD model. We further confirm the prosurvival role of *Akt1 *and *JNK *in DA neurons against PQ with evaluation of delayed DA neuron loss with overexpression of wild type Akt and JNK in DA neurons. However, addition of MC failed to further prevent the DA neuron loss in the Akt over-expressed DA neurons against PQ. These neuron count data parallel the survival data obtained with the flies over-expressed with wild type Akt and JNK against PQ and PQ with MC. MC improved the survival of the flies over-expressed with wild type Akt and JNK in the same proportional as in wild type (control) flies against PQ.

 We found that heterozygous loss-of-function mutant ERK/*rolled* lacks any detectable functional involvement in our model, although the result could also infer the lack of sufficient knockdown due to the heterozygosity of the strain. Recently, genetic interaction between *Drosophila *DJ-1 and Ras/ERK but not with PI3K/Akt thereby indicating that the prosurvival effect of DJ-1 is mediated via ERK in DA neurons [55]. While the lack of effect in our examination of an *ERK/rolled *mutant may be explained, as noted above, by insufficient reduction of function, this may also be an instance in which the cellular effects of PQ and mutations in PD genes may diverge. Future studies will address this point.

Finally, we analyzed the role of programmed cell death in PQ neurotoxicity by testing heterozygous mutants for the pro-apoptotic gene, *reaper*, and the programmed cell death initiator, caspase-9 ortholog, *dronc*. We found that a heterozygous loss-of-function *reaper *mutant displayed comparable survival to wild type flies on PQ, while *dronc *mutants showed an increase in lifespan. PQ has been shown to induce apoptosis via activation of caspases 3 and 9 and inhibition of Bcl-2 family members except Bax [56]. Thus, our results for *dronc* suggest a parallel mode of action in flies and mammals.

 In conclusion, in addition to our finding of protective mechanisms of MC against PQ in this PD model and novel MC-*Punch *mutant interactions, our results here provide *in vivo *evidence for essential roles for stress-responsive kinases in the response to PQ. In this paper, we present data showing the failure of heterozygous *Akt1* and *JNK* loss-of-function mutants to respond to MC. However, ingestion of 10 mM MC by flies over-expressing wild type Akt1 and JNK in DA neurons does not provide additional levels of protection against PQ. Thus, these pathways may not be directly affected by MC or, alternatively, there may be an upper limit above which these kinases can no longer be effective as damage from continuous PQ exposure in these acute toxin model accumulates. Further exploration of the roles of these pathways in various mutant backgrounds in chronic or sporadic exposure models of the effects of PQ will be productive avenues to follow as will analyses of tissue-specific Akt and JNK responses.
